# Long-Term Outcomes of Extent of Revascularization in Complex High Risk and Indicated Patients Undergoing Impella-Protected Percutaneous Coronary Intervention: Report from the Roma-Verona Registry

**DOI:** 10.1155/2019/5243913

**Published:** 2019-04-09

**Authors:** Francesco Burzotta, Giulio Russo, Flavio Ribichini, Anna Piccoli, Domenico D'Amario, Lazzaro Paraggio, Leonardo Previ, Gabriele Pesarini, Italo Porto, Antonio Maria Leone, Giampaolo Niccoli, Cristina Aurigemma, Diana Verdirosi, Filippo Crea, Carlo Trani

**Affiliations:** ^1^Fondazione Policlinico Universitario A. Gemelli IRCCS, Roma, Italy; ^2^Università Cattolica del Sacro Cuore, Roma, Italy; ^3^Division of Cardiology, Department of Medicine, University of Verona, Verona, Italy

## Abstract

**Objective:**

To investigate the effect of extent of revascularization in complex high-risk indicated patients (CHIP) undergoing Impella-protected percutaneous coronary intervention (PCI).

**Background:**

Complete revascularization has been shown to be associated with improved outcomes. However, the impact of more complete revascularization during Impella-protected PCI in CHIP has not been reported.

**Methods:**

A total of 86 CHIP undergoing elective PCI with Impella 2.5 or Impella CP between April 2007 and December 2016 from 2 high volume Italian centers were included. Baseline, procedural, and clinical outcomes data were collected retrospectively. Completeness of coronary revascularization was assessed using the British Cardiovascular Intervention Society myocardial jeopardy score (BCIS-JS) derived revascularization index (RI). The primary end-point was all-cause mortality. A multivariate regression model was used to identify independent predictors of mortality.

**Results:**

All patients had multivessel disease and were considered unsuitable for surgery. At baseline, 44% had left main disease, 78% had LVEF ≤ 35%, and mean BCIS-JS score was 10±2. The mean BCIS-JS derived RI was 0.7±0.2 and procedural complications were uncommon. At 14-month follow-up, all-cause mortality was 10.5%. At follow-up, 67.4% of CHIP had LVEF ≥ 35% compared to 22.1% before Impella protected-PCI. Higher BCIS-JS RI was significantly associated with LVEF improvement (p=0.002). BCIS-JS RI of ≤ 0.8 (HR 0.11, 95% CI 0.01- 0.92, and p = 0.042) was an independent predictor of mortality.

**Conclusions:**

These results support the practice of percutaneous Impella use for protected PCI in CHIP. A more complete revascularization was associated with significant LVEF improvement and survival.

## 1. Introduction

Patients with poor left ventricular (LV) function and complex coronary anatomy (such as multivessel disease, left main disease, and last remaining vessel) may be unsuitable for surgical revascularization due to high risk for periprocedural complications [1]. Percutaneous coronary intervention (PCI) is an alternative revascularization strategy in such patients, commonly referred to as complex high-risk indicated patients (CHIP) [2]. The use of percutaneous left ventricular assist devices may minimize the risk of hemodynamic compromise during such high risk PCI and allow complete revascularization, thus improving outcomes. The feasibility, safety, and hemodynamic effects of Impella devices during high-risk PCI have been demonstrated in the PROTECT-I and PROTECT-II trials and in multiple real-world studies [3–8].

Recent meta-analysis by Pasceri et al. suggests improved outcomes following complete revascularization in patients with multivessel disease [9]. Importantly, studies suggest the possibility of complete revascularization during Impella-protected PCI, resulting in reduced need for repeat revascularizations [10]. The purpose of the present study was to investigate the effect of extent of revascularization, as assessed using the BCIS-JS, in CHIP undergoing Impella-protected PCI in an all comers cohort from two high volume Italian centers.

## 2. Methods

### 2.1. Study Population

**Figure 1:**
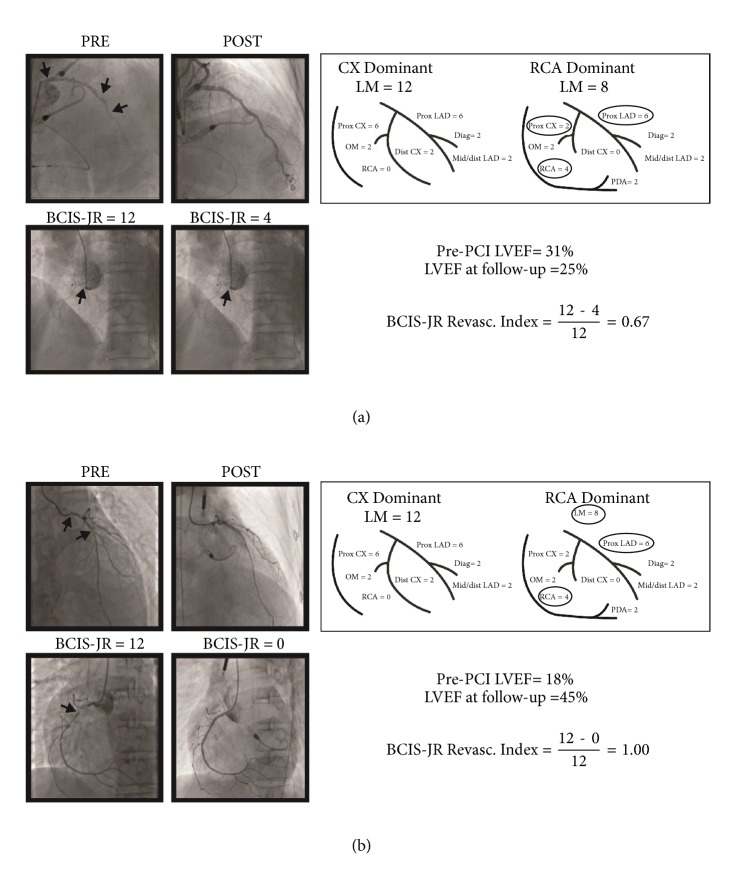
*British-Cardiovascular-Intervention-Society jeopardy-score (BCIS-JS) calculation examples in a patient with complete (a) and in a patient with incomplete (b) myocardial revascularization*. Patient in (a) showed a significant improvement of left ventricular function, while patient in (b) died after 6 months of follow-up. Arrows indicate significant coronary stenosis. PCI=percutaneous coronary intervention and LVEF=left ventricular ejection fraction.

This is a retrospective study using a prospectively maintained database: data of all consecutive patients undergoing elective Impella-protected PCI from April 2007 to December 2016 in two high-volume (>1000 PCI per year) Italian cardiac catheterization laboratories. Patients with cardiogenic shock and acute myocardial infarction within 24 hours were excluded. According to the hospital practice at the two centers, the need for Impella-protected high-risk PCI was assessed based on collegial heart-team discussion in which the potential benefit of myocardial revascularization was agreed and surgical revascularization was considered not feasible. Coronary stenosis ≥70% was considered significant (≥50% in case of left main coronary artery lesion) by visual assessment on angiogram. Alternatively, it was assessed by guided by fractional flow reserve (FFR) or by stress test imaging. All patients provided written consent to undergo PCI with Impella support after detailed explanation of the procedural features. Clinical data, operative risk score (EuroSCORE I), and procedural data were prospectively collected. Synergy between Percutaneous Coronary Intervention with TAXUS and Cardiac Surgery (SYNTAX) score before and after the procedure was calculated for all patients without previous coronary surgery. For the present study, an interventional cardiology fellow (blinded to patient's clinical presentation and outcome) graded the myocardium at jeopardy before and after PCI using the British Cardiovascular Intervention Society (BCIS) Jeopardy Score (JS) algorithm [11]. Extent of revascularization was assessed using the BCIS-JS revascularization index (RI): (BCIS-JS_pre_ -BCIS-JS_post_)/ BCIS-JS_pre_ as previously reported [12]. Two examples of BCIS-JS RI calculations are provided in Figures 1(a) and 1(b).

### 2.2. Impella-Protected PCI

The Impella 2.5 or Impella CP (after its release in Italy) heart pumps were used and implantation was performed through percutaneous trans-femoral approach. All patients received iliac-femoral artery axis assessment by ultrasonography and/or peripheral angiography during a pre-PCI work-up. All femoral punctures were fluoroscopy-guided. Prior to Impella sheath placement, peripheral angiography (through the radial or contralateral femoral access selected for PCI) was performed to confirm the suitability of iliac-femoral arterial axis anatomy. Accordingly, when atherosclerotic burden or tortuosity was high, the contralateral iliac-femoral axis was assessed and the most favorable side was chosen for Impella implantation. The presence of atherosclerotic disease of the iliac-femoral axis with nonsignificant (<50% diameter) stenosis was not considered an exclusion criterion. No failure of device implantation was reported due to the screening process including systematic iliac-femoral axis assessment.

After femoral artery stick, a 6-8 Fr sheath was inserted. Then, “pre-closure” technique with suture-based hemostatic devices was used. The “pre-closure” technique was performed with (according to operator's yield) the Prostar XL10F or Perclose ProGlide devices (Abbott Vascular Devices, Redwood City, CA, USA) and consisted of the suture deployment before introduction of the (13 Fr or 14 Fr) Impella 2.5 or CP sheath. After the procedure, Impella was removed and the sutures were tied by pushing down knot(s) in order to achieve percutaneous hemostasis. Of note, when using ProGlide, a double ProGlide pre-closure technique was adopted, based on sequential insertion of two ProGlide devices rotated in opposite sides 30-45°, to create an interrupted X-figure closure [13]. After dedicated sheath insertion, a 6 Fr diagnostic catheter (Judkins right or pigtail) was advanced into the LV and used to place the 300 cm extra-support guidewire into the LV. Then, the Impella catheter was advanced over the guidewire through the aortic valve into the LV. Impella was then activated after removal of the guidewire and LV assistance maintained throughout the procedure. Our center started using the pre-closure technique before 2007 and gaining since then experience in the management of vascular access with large bore devices resulting in low vascular complication rate (2%) in our registry. Our experience with double pre-closure system using Perclose has already been reported elsewhere [14]. In more recent years, our puncture technique has undergone further improvements by combining angiographic and ultrasound guidance [15, 16].

PCI was performed by the radial or (in patients with unsuitable radial accesses) by contralateral femoral approach using 6-8 Fr guiding catheters. Selection of guidewires, balloons, and stents was based operator's choice. Drug-eluting stent implantation was the main PCI technique and debulking with Rotablator was the main adjunctive device used for severely calcified coronary segments. At the end of PCI, Impella speed was gradually decreased and patient's hemodynamic condition was evaluated. In case of hemodynamic stability, Impella was immediately removed. In the case of mechanical hemostasis failures, manual compression followed by compressive bandage was adopted. Of note, access-artery angiography to confirm hemostasis and to rule-out vascular complications was systematically performed. In all patients, heparin was administered (initial weight-adjusted intravenous bolus then further boluses administered in order to keep the activated clotting time between 250 and 300s) and double antiplatelet therapy was started before PCI initiation and recommended for 12 months. Blood samples were obtained at 6 and 24 h after the procedure to measure hemoglobin and creatinine levels. Additional laboratory exams were performed if clinically indicated. The occurrence of any complication during the hospital stay was prospectively recorded into the catheterization laboratory database.

### 2.3. Procedural and Clinical Outcome Assessment

For the present study, clinical records were carefully evaluated and clinical follow-up was obtained by reviewing the outpatient visit reports or by telephone interview. Access site or bleeding complications were classified according to the Valve Academic Research Consortium (VARC) and the Bleeding Academic Research Consortium (BARC) criteria [17, 18]. The primary study end-point was all-cause mortality. Major adverse cardiac and cerebrovascular events (MACCE) were defined as the composite of death and/or acute myocardial infarction (AMI) or target vessel revascularization (either percutaneous or surgical) or stroke. AMI during follow-up was defined as the rise and fall of cardiac enzymes (usually serum high-sensitivity Troponin I) in the presence of electrocardiogram signs or symptoms compatible with myocardial ischemia, as described in the third universal definition of myocardial infarction [19]. Stroke was defined as any new, permanent, global, or focal neurological deficit ascertained by a standard neurological examination, lasting longer than 24 hours or less if evidence of cerebral infarction was obtained by imaging. In our study renal failure was defined per the Kidney Disease Improving Global Outcomes (KDIGO) guidelines as any abnormality of kidney function (decreased glomerular filtration rate <60 ml/min/1.73m^2^) or kidney structure (e.g., kidney transplantation).

### 2.4. Left Ventricular Function

Echocardiographic assessment of the LV ejection fraction (LVEF), aortic valve, and the presence of intraventricular thrombus was systematically performed before the procedure. After the index hospitalization, clinical follow-up (with echocardiographic examination) was systematically recommended. Echocardiographic assessment of LV function was performed according to the biplane method of disk summation (modified Simpson's rule) [20]. Changes in LVEF between baseline and post-PCI (at the longest available echocardiographic examination) were estimated by calculating the “indexed” LVEF variation, calculated as (LVEF_pre-PCI_-LVEF_post-PCI_)/LVEF_pre-PCI_.

### 2.5. Statistical Analysis

Continuous variables are presented as mean with standard deviation (SD) and categorical variables as numbers and percentages. The continuous variables related to LVEF and BCIS-JS RI were categorised in tertiles. Comparisons of continuous variables across different groups were performed using Student t test or ANOVA test (as appropriate). Categorical variables were evaluated using *χ*^2^ test or Fisher's exact test, as appropriate. Comparisons between pre-PCI and post-PCI data were performed using paired t-test. A Cox regression analysis was performed to identify the independent predictors of mortality among the main baseline characteristics (age, gender, diabetes, renal failure, prior myocardial infarction, prior PCI, prior cardiac surgery, clinical presentation, advanced New York Heart Association class, EuroSCORE, and BCIS-JS RI). Adjusted hazard ratios (HR) with associated 95% confidence interval (CI) were calculated for the significant mortality predictors and corresponding adjusted survival curves were determined. A 2-tailed, p-value <0.05 was established as the level of statistical significance. All statistical analyses were performed using SPSS software v22.0 (IBM Corporation, Armonk, New York).

## 3. Results

### 3.1. Patient Population

From April 2007 to December 2016, a total of 86 patients (mean age 72±10 years, 91.8% men) underwent Impella protected high-risk PCI at two high volume Italian centers and were included in the present analysis. Baseline characteristics of patients are listed in Table 1. Patients were highly symptomatic with New York Heart Association class III or IV in 69.7%. They had high prevalence of hypertension (78%), dyslipidemia (62%), diabetes (44%), previous myocardial infarction (35%), and previous coronary bypass grafting surgery (22%). About 58% of patients were admitted for a non-ST elevation myocardial infarction (NSTEMI). About 78% had LVEF < 35% (mean 31±9%). All patients had multivessel disease and 44% had left main disease. The mean SYNTAX score was 31±10 and mean EuroSCORE I was 9±3. The extent of jeopardized myocardium was large with mean pre-PCI BCIS-JS of 10 (score range: 0-12). All patients were deemed unsuitable for surgery on the basis of heart-team discussion. The baseline characteristics of the present study cohort were listed alongside previously published clinical studies: PROTECT II trial, Europella, and USpella registries [4–6] (Table 1).

### 3.2. Procedural and Safety Outcomes

Seventy-four patients (86%) received hemodynamic support with Impella 2.5 and 12 patients (14%) with Impella CP (Table 2). In majority of patients (77%), PCI was performed via the radial artery access. Thirty-five (41%) patients underwent left main PCI and almost three-quarters of patients were treated on at least two vessels including bifurcations and diffusely diseased vessels (Table 2). Thirteen patients (15%) received rotational atherectomy for heavily calcified lesions. At least one drug eluting stent was implanted in 83 (96%) patients, and the remaining 3 (4%) patients received bare metal stents due to concerns regarding their tolerance for prolonged dual antiplatelet therapy. Both SYNTAX score and BCIS-JS were significantly reduced after PCI compared to baseline (from 31±10 to 12±9 and from 10±2 to 3±3 respectively, p < 0.001 for both). Patients had complete revascularization as suggested by mean BCIS-JS revascularization index of 0.7±0.2. Successful hemostasis was achieved by insertion of double preimplanted Perclose ProGlide in 63%, Prostar XL in 12%, and manual compression in 25%.

Bleeding and vascular complications occurred in 14% and 2%, respectively (Table 3). There were 6 cases of minor hematomas that required no specific intervention (BARC Type I), 2 patients required bail-out balloon peripheral angioplasty to facilitate hemostasis by manual-compression (BARC Type II), and 4 patients had decrease in hemoglobin by >3 g/dL with three of them requiring blood transfusion (BARC Type III). Two minor vascular complications occurred. One patient had distal embolization few hours after PCI (acute lower limb ischemia plus occlusion of lower limb arteries based on ultrasound examination) and was successfully managed by urgent peripheral angioplasty on tibial-peroneal arteries. The other case was an access site vascular injury consisting of angiographically documented common femoral artery occlusion after Impella 2.5 pump removal. Since the patient was asymptomatic, and collateral branches provided full distal supply (probably due to pre-existing superficial femoral artery disease), a conservative management was selected, and the clinical course was uneventful. No other main procedure-related complication was noticed and a total of 94% of patients were discharged alive (see below for death reports).

In our study the median value for the duration of Impella support was 104 minutes (range 55-3151) and Impella malfunctions were not reported. Further insights into procedural hemodynamic behavior and Impella pump performance were available only in 37 patients [21].

### 3.3. Left Ventricular Function

**Figure 2:**
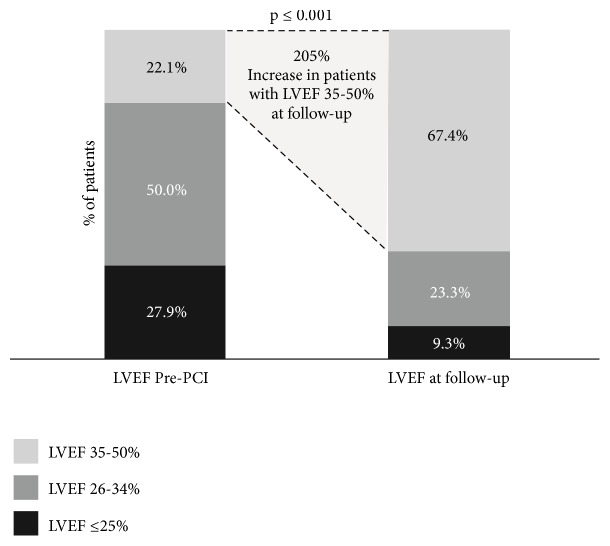
*Left ventricular ejection fraction improvement during the follow-up after IMP-protected PCI*. The figure shows the comparison of left ventricular ejection fraction impairment between pre-PCI and follow-up assessment. LVEF=left ventricular ejection fraction and PCI=percutaneous coronary intervention.

Seventy-nine patients (92%) underwent echocardiography at mean follow-up of 6 months (range 1-12 months) and significant improvement in LVEF was observed (31±9% at baseline to 39±9%, p < 0.001). Of the 79 patients, 27.9% presented with pre-PCI LVEF ≤ 25%, 50% with LVEF of 26-34%, and 22.1% with LVEF of 35-50%. After PCI, only 9.3% had LVEF ≤ 25%, and 23.3% had LVEF of 26-34%. Patients had significant improvement in LVEF post-Impella-protected PCI with 67.4% having LVEF of 35-50% (Figure 2).

**Figure 3:**
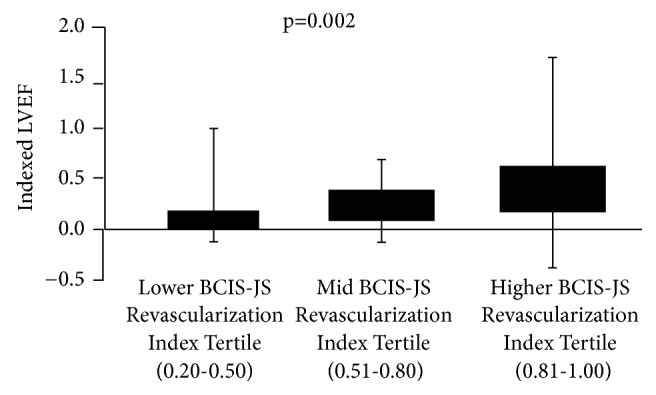
*Left ventricular ejection fraction improvement according to revascularization extent*. The figure shows the left ventricular improvement in patients stratified according to revascularization extent as evaluated by the British Cardiovascular Intervention Society Jeopardy Score (BCIS-JS) tertiles. LVEF=left ventricular ejection fraction.

Among the different preprocedural and procedural factors, pre-PCI LVEF (p < 0.001) and BCIS-JS revascularization index (p < 0.001) significantly influenced “indexed” LVEF variation. In particular, significantly higher indexed LVEF variation was observed in patients with higher BCIS-JS revascularization index (p = 0.002) (Figure 3).

### 3.4. Clinical Outcome and Its Determinants

The rate of major adverse cardiac and cerebrovascular events (MACCE) during mean follow-up of 14 months was 24% (Table 4). A total of nine deaths (10.5%) occurred during the study. In particular, one death occurred few hours after procedure due to acute bare metal stent thrombosis. Four deaths occurred within 30 days from the index procedure: three patients died of progressive respiratory failure in the presence of refractory heart failure and severe chronic obstructive pulmonary disease and one patient (exhibiting persistent electrical instability) died during ventricular tachycardia ablation attempt. Among the remaining four patients who died after 30 days, one had fatal stent thrombosis at 6-months, and three had progressive congestive heart failure. Repeat PCI was performed in 12 patients (14%) and CABG in 1 patient (1%). AMI occurred in 6 patients (7%) and no case of stroke was reported.

**Figure 4:**
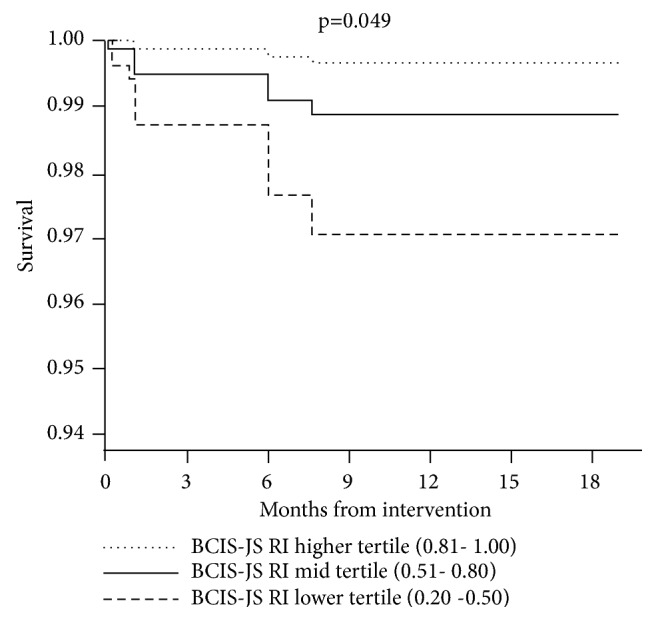
*Survival curves according to revascularization extent*. The figure shows the adjusted survival curves in the study population stratified according to British Cardiovascular Intervention Society Jeopardy Score (BCIS-JS) revascularization index tertiles. RI= revascularization index.

In the multivariate analysis including all the main baseline characteristics, EuroSCORE I > 11 (HR 311.8, 95% CI 10.9 to 8952, and p = 0.001) and BCIS-JS revascularization index of ≤ 0.8 (HR 0.11, 95% CI 0.01 to 0.92, and p = 0.042) were the only independent predictors of mortality (Table 5). No significant association was found with residual Syntax Score. Based on Kaplan-Meier analysis, patients with more complete revascularization of angiographically significant stenosis (RI > 0.8 to 1.0) had a survival advantage, both early after PCI and in the longer term, compared to those with less revascularization (RI 0.2 to 0.5 or 0.51 to 0.80; p = 0.049) (Figure 4).

## 4. Discussion

In this large cohort of patients treated by IMP-protected PCI in two experienced Italian centers we found that:

1. Impella-protected PCI in CHIP is associated with LVEF recovery and very promising survival rates
2. the extent of the coronary revascularization achieved during IMP-protected PCI in CHIP is associated with LVEF recovery and survival.

Collectively, the results of our study suggest that Impella-protected PCI is an attractive revascularization strategy in CHIP, leading to favorable outcomes.

All the patients in our study had multivessel disease, were deemed to be ineligible for surgery, and had higher prevalence of left main disease as compared to the patients randomized in the PROTECT-II trial (Table 1), reflecting higher complexity and mortality of patients in routine clinical practice. The study by Waldo et al. suggested that surgical ineligibility is common and associated with higher mortality among patients with multivessel or left main disease undergoing nonemergent PCI [22]. Despite the high risk of mortality, the survival rate in our study was favorable at 90% likely due to more complete revascularization with Impella-protected PCI. In fact, BCIS-JS derived RI was an independent predictor of mortality in multivariate analysis.

The present study assessed the extent of revascularization using the BCIS-JS, which takes into account the entire myocardial area at risk [12]. The mean BCIS-JS-derived RI of 0.7 demonstrates the utility of Impella-protected PCI in achieving more complete revascularization. The finding of higher survival with higher RI is in line with previous study demonstrating survival benefit following complete revascularization and the prognostic value of BCIS-JS in predicting mortality in high-risk PCI [12]. Complete revascularization during Impella-protected PCI is likely due to the greater hemodynamic stability provided by Impella, thus allowing operators to perform more vigorous procedures including rotational atherectomy for heavily calcified lesions [4]. Généreux et al. [23] had previously shown that higher residual Syntax Score was associated with poor short- and long-term prognosis in patients with moderate/high risk acute coronary syndrome. However, their study population was slightly different from that presented in this study: they had a lower baseline Syntax Score and only a minority of patients presented with reduced LV function. While Syntax Score remains the main tool for risk assessment according to coronary anatomy complexity our results suggest that BCIS-JS score and the BCIS-JS derived RI might provide a better risk evaluation in the work-up of CHIP.

More complete revascularization with Impella-protected PCI resulted in improvement in cardiac function with about 70% of CHIP having LVEF ≥ 35%. Moreover, BCIS-JS derived RI significantly influenced “indexed” LVEF variation. This is a novel finding as previous studies of Impella in high risk PCI have inferred more complete revascularization based on reduced need for repeat revascularization [4, 10]. As demonstrated in previous studies with STEMI and multivessel disease, complete revascularization was associated with improved survival outcomes and complications [9, 24], although our patient population included 58% with non-STEMI.

Previous studies have documented the safety of Impella use in high-risk PCI at 30 and 90 days [4, 25]. A substudy of the PROTECT II trial [26] focused on patients with three-vessel coronary artery disease and reduced LV function, showing improved outcomes when Impella 2.5 was used as LV support device, compared to intra-aortic balloon pump (IABP). The composite endpoint of major cardiac events was significantly lower in the Impella group compared to IABP group (32.9% vs 42.4% at 30 days and 39.5% vs 51.0% after 90 days of follow-up). However, all-cause death rates, for which the study was not powered, were similar between the two groups. Moreover, data regarding Impella supported PCI in patients undergoing unprotected left main (LM) revascularization have been extrapolated from the USpella registry [27] demonstrating its feasibility and safety. Although the analysis was mainly based on patients undergoing LM PCI, they achieved nearly complete revascularization with low procedural risk.

Our results demonstrate that protected PCI with Impella 2.5 in CHIP yields acceptable complication rates with a MACCE rate of 24% at longest follow-up of 14 months. Furthermore, periprocedural adverse events occurred less frequently given the uniform adoption of best practices including careful iliac-femoral axis selection, meticulous femoral puncture, high radial access use for PCI, early pump removal with angiographic hemostasis check, use of suture-based devices preclosure technique, and endovascular management of vascular complications.

Currently, the role of Impella in the setting of high-risk procedures has been addressed in expert consensus documents [1]. The role of mechanical circulatory support is only minimally addressed in the guidelines, due to lack of strong evidence. The 2011 ACCF/AHA/SCAI guidelines [28] state that elective insertion of an appropriate hemodynamic support device as an adjunct to PCI may be reasonable in carefully selected high-risk patients (class IIb, level of evidence C). The 2010 ESC guidelines [29] only suggest that circulatory support should be considered in nonemergent high-risk PCI procedures—such as for LM disease, single remaining patent coronary artery, and complex chronic total occlusions—performed by adequately experienced operators at centres that have access to circulatory support and on site cardiovascular surgery. No indications for specific devices (i.e., Impella, ECMO) are given.

Indeed, more data about Impella supported PCI in the setting of high-risk patients are needed. However, this study provides evidence supporting the use of Impella in CHIP.

### 4.1. Limitations

Given the observational and retrospective nature of our study, the findings should be considered hypothesis generating. Randomized trials are required to fully validate the findings and establish causality. This is a study from two centers in Italy with unique geographic and demographic characteristics and therefore results may not be generalizable.

### 4.2. Conclusion

Impella-protected PCI in CHIP is associated with LVEF improvement. A more complete myocardial revascularization was associated with LVEF improvement and better survival.

## Figures and Tables

**Table 1:** Baseline characteristics of study population.

	Present Study	PROTECT II	EUROpella	USpella
Number of enrolling centres	2	112	10	18
Number of enrolled patients	86	225*∗*	144	175
Age (years), mean±SD	72±10	68±11	72±10	70±10
Gender, males/females	79/7	180/45	117/27	74/101
*Cardiovascular Risk Factors, n(*%)				
Hypertension	67 (78)	/	97 (67)	/
Dyslipidemia	53 (62)	/	93 (65)	/
Diabetes	38 (44)	117 (52)	62 (43)	82 (47)
Smoking	16 (19)	/	61 (42)	/
Family history of CAD	16 (19)	/	/	/
Renal Failure°	27 (31)	102 (23)	41 (28)	58 (33)
*Past Cardiac History, n(*%)				
Prior MI	30 (35)	/	76 (53)	98 (56)
Prior PCI	14 (16)	/	/	84 (48)
Prior CABG	19 (22)	85 (38)	42 (29)	49 (28)
*Clinical presentation, n(*%)				
STEMI	13 (15)	/	/	/
NSTEMI	50 (58)	55 (37)	/	/
SA	23 (27)	93 (63)	/	/
*NYHA III-IV, n(*%)	60 (70)	151 (67)	/	115 (66)
*LVEF, mean±SD (*%)	31±9	23±6	/	31±17
*LVEF ≤35*%*, n(*%)	67 (78)	216 (100)	92 (64)†	121 (69)
*EuroSCORE,* mean±SD	9±3	9±6	8±3	/
Lower Tertile (3-7)	30 (35)			
Mid Tertile 2 (8-10)	30 (35)			
Higher Tertile 3 (11-20)	26 (30)			
*Unsuitable for surgery ‡, n (*%)	86 (100)	144 (64)	62 (43)	98 (56)
*Angiographic characteristics*				
Multivessel disease, n (%)	86 (100)	/	118 (82)	155 (89)
Left main disease, n (%)	38 (44)	18 (8)	76 (53) ^§^	89(51)
Syntax Score	31±10	30±13	/	37±16
BCIS-JS	10±2	/	/	/

*∗* Randomized Impella arm.

† LVEF <40%

‡ On the basis of heart-team discussion.

^§^PCI on left main coronary artery.

° Abnormalities of kidney function (decreased glomerular filtration rate <60 ml/min/1.73m2) or kidney structure (e.g., kidney transplantation).

CAD=coronary artery disease; MI=myocardial infarction; PCI=percutaneous coronary intervention; CABG=coronary artery bypass graft; STEMI=ST elevation myocardial infarction; NSTEMI=non-ST elevation myocardial infarction; SA= stable angina; NYHA=New York Heart Association; LVEF=left ventricular ejection fraction; BCIS-JS= The British Cardiovascular Intervention Society myocardial Jeopardy Score.

**Table 2:** Procedural characteristics.

CHARACTERISTICS	N=86 (%)
*Approach for PCI*	
Radial	66 (77)
Femoral	20 (23)
*Number of treated vessels*	
One-vessel PCI	23 (27)
Two-vessel PCI	39 (45)
Three-vessel PCI	24 (28)
PCI on bifurcation	52 (60)
PCI with Rotablator	13 (15)
At least one DES implanted	83 (96)
*Post-PCI Angiographic scores *	
Syntax Score	12±8.7^*∗*^
BCIS-JS	3±3^*∗*^
*BCIS-JS Revascularization Index*, mean±SD	0.7±0.2
Lower Tertile (0.20-0.50)	28 (33)
Mid Tertile (0.51-0.80)	29 (34)
Higher Tertile (0.81-1.00)	29 (34)
*Impella pump*	
2.5	74 (86)
CP	12 (14)
*Impella support duration*, min°	104 (55-3151)
*Haemostasis technique*	
Double Perclose	54 (63)
Prostar	10 (12)
Manual compression	22 (25)

^*∗*^ p<0.001 as compared with baseline values.

° value expressed as median with range.

PCI= percutaneous coronary intervention; DES=drug eluting stent; BCIS-JS= the British Cardiovascular Intervention Society myocardial Jeopardy Score.

**Table 3:** Periprocedural bleeding and vascular complications.

COMPLICATION	N=86 (%)
*Bleedings (BARC criteria)*	*12 (14)*
Type I	6 (7)
Type II	2 (2)
Type III	4 (5)
Type IV	0
Type V	0
*Vascular complications (VARC criteria)*	2 (2)
Major vascular complication	0
Minor vascular complication	2
Percutaneous closure device failure	0

BARC= Bleeding Academic Research Consortium; VARC= Valve Academic Research Consortium; see text for detailed description of complications.

**Table 4:** Clinical outcome at longest follow-up.

ADVERSE EVENT	Present Study N=86 (%)	PROTECT II N=225*∗* (%)°	EUROpella N=144 (%)°	USpella N=175 (%)°
MACCE	21 (24)	90 (41) ^§^	17 (12)	
Re-PCI	12 (14)	8 (4)	/	1 (1)
CABG	1 (1)	2 (1)	0	^†^
AMI	6 (7)	27 (12)	0	2 (1)
Stroke	0	2 (1)	1 (1)	1 (1)
*All-cause death *	9 (10)	27 (12)	8 (6)	7 (4)

^*∗*^ Randomized Impella arm

° Data at longest follow up available in the intention-to-treat population

^§^ Composite rate of intra- and postprocedural major adverse events (MAEs) at discharge or 30-day follow-up, whichever was longer. The composite primary end point components included all-cause death, Q-wave or non–Q wave

MI, stroke, or transient ischemic attack, any repeat revascularization procedure, need for a cardiac or a vascular operation, acute renal insufficiency, severe intraprocedural hypotension requiring therapy, cardiopulmonary resuscitation or ventricular tachycardia requiring cardioversion, aortic insufficiency, and angiographic failure of PCI.

^†^ Includes PCI or CABG revascularization.

MACCE=major adverse cardiac and cerebrovascular events; PCI= percutaneous coronary intervention; CABG=coronary artery bypass grafting; AMI=acute myocardial infarction

**Table 5:** Multivariate analysis for independent predictors of mortality.

	P-value	Adjusted HR (95% CI)
*EuroSCORE*		
mid tertile vs. lower tertile	0.053	31.4 (1.0-1036.8)
higher tertile vs. lower tertile	0.001	311.8 (10.9-8952.0)
*BCIS-JS Revascularization Index*		
mid tertile vs. lower tertile	0.490	0.38 (0.03-5.83)
higher tertile vs. lower tertile	0.042	0.11 (0.01-0.92)

Other variables this model controlled for: age, gender, diabetes, renal failure, prior MI, prior CABG, NSTEMI, and SA.

MI= myocardial infarction; PCI= percutaneous coronary intervention; CABG=coronary artery bypass grafting; NSTEMI=non ST elevation myocardial infarction; SA= stable angina; NYHA=New York Heart Association; BCIS-JS= the British Cardiovascular Intervention Society myocardial Jeopardy Score.

## Data Availability

The clinical and procedural data used to support the findings of this study are included within the article.
